# The Vpr protein from HIV-1: distinct roles along the viral life cycle

**DOI:** 10.1186/1742-4690-2-11

**Published:** 2005-02-22

**Authors:** Erwann Le Rouzic, Serge Benichou

**Affiliations:** 1 Institut Cochin, Department of Infectious Diseases, INSERM U567, CNRS UMR8104, Université Paris 5, Paris, France

## Abstract

The genomes of human and simian immunodeficiency viruses (HIV and SIV) encode the gag, pol and env genes and contain at least six supplementary open reading frames termed *tat*, *rev*, *nef*, *vif*, *vpr*, *vpx *and *vpu*. While the *tat *and *rev *genes encode regulatory proteins absolutely required for virus replication, *nef*, *vif*, *vpr, vpx *and *vpu *encode for small proteins referred to "auxiliary" (or "accessory"), since their expression is usually dispensable for virus growth in many *in vitro *systems. However, these auxiliary proteins are essential for viral replication and pathogenesis *in vivo*. The two *vpr*- and *vpx*-related genes are found only in members of the HIV-2/SIVsm/SIVmac group, whereas primate lentiviruses from other lineages (HIV-1, SIVcpz, SIVagm, SIVmnd and SIVsyk) contain a single *vpr *gene. In this review, we will mainly focus on *vpr *from HIV-1 and discuss the most recent developments in our understanding of Vpr functions and its role during the virus replication cycle.

## Introduction

**Figure 1:**
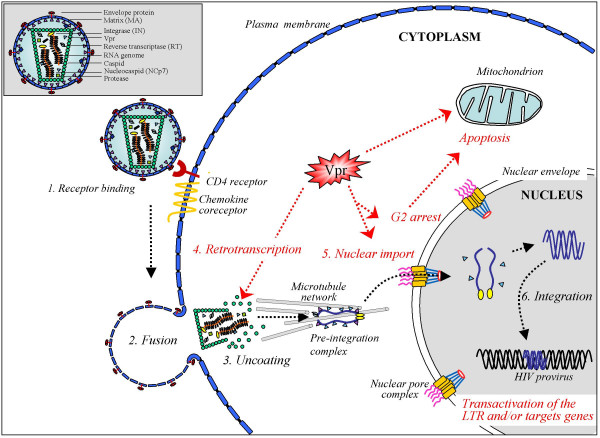
Schematic view of the early steps of the HIV-1 infection of a target cell. The functional events in which the Vpr protein is involved are highlighted. Vpr has been shown to play multiple functions during the virus life cycle, including an effect on the accuracy of the reverse-transcription process, the nuclear import of the viral DNA as a component of the pre-integration complex, cell cycle progression, regulation of apoptosis, and the transactivation of the HIV-LTR as well as host cell genes.

The viral protein R (Vpr) of HIV-1 is a small basic protein (14 kDa) of 96 amino acids, and is well conserved in HIV-1, HIV-2 and SIV [1]. The role of Vpr in the pathogenesis of AIDS is undeniable, but its real functions during the natural course of infection are still subject to debate. The Vpr role in the pathophysiology of AIDS has been investigated in rhesus monkeys experimentally infected with SIVmac, and it was initially shown that monkeys infected with a *vpr *null SIV mutant decreased virus replication and delayed disease progression [2,3]. Moreover, monkeys infected with a SIV that did not express the *vpr *and *vpx *genes displayed a very low virus burden and did not develop immunodeficiency disease [4,5]. Regarding these *in vivo *phenotypic effects, numerous laboratories have dissected the role of Vpr in various *in vitro*, *in vivo *and *ex vivo *systems to explore the contribution of this protein in the different steps of the virus life cycle. Despite its small size, Vpr has been shown to play multiple functions during virus replication, including an effect on the accuracy of the reverse-transcription process, the nuclear import of the viral DNA as a component of the pre-integration complex (PIC), cell cycle progression, regulation of apoptosis, and the transactivation of the HIV-LTR as well as host cell genes (Fig. 1). Furthermore, Vpr is found in virions, in cells, and exists as free molecules found in the sera and the cerebrospinal fluid of AIDS patients, indicating that it may exert its biological functions through different manners.

### Structure of the HIV-1 Vpr protein

**Figure 2:**
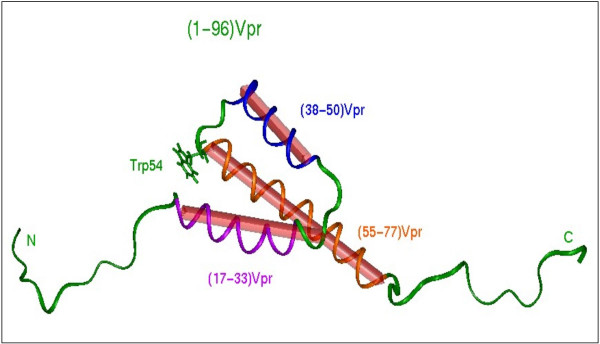
Three-dimensional structure of the HIV-1 Vpr protein (from [15]). The three α-helices (17–33, 38–50, 55–77) are colored in pink, blue and orange, respectively; the loops and flexible domains are in green. We can the Trp54 residue localized between the second and the third a-helix, and that is likely accessible for protein-protein interaction with UNG2 [54].

Because the full length protein aggregated in aqueous solution, the overall structure of Vpr has been difficult to access [6], and preliminary strategies used two distinct synthetic peptides corresponding to Vpr (1–51) and (52–96) fragments for NMR and circular dichroism studies [6-9]. As previously predicted [10], the structure of the Vpr(1–51) fragment has a long motif of α helix turn-α helix type encompassing the Asp17-Ile46 region, and ends with a γ turn [8]. The Vpr(52–96) fragment contains an α-helix encompassing the 53–78 region that is rich in leucine residues [7]. One side of the helix offers a stretch of hydrophobic residues that can form a leucine-zipper like motif [11]. This structure may account for the formation of Vpr dimers [7,12,13] and/or for the interaction with cellular partners [14]. Finally, NMR analysis of a soluble full length Vpr (1–96) polypeptide was recently performed, and gave access to the tertiary structure of the protein (Fig. 2), confirming the amphipathic nature of the three α-helices of HIV-1 Vpr. The helices are connected by loops and are folded around a hydrophobic core [15] surrounded by a flexible N-terminal domain and a C-terminal arginine-rich region that are negatively and positively charged, respectively. Four conserved prolines (positions 5, 10, 14 and 35) which present *cis/trans *isomerization are found in the N-terminal domain [16]. It was reported that the cellular peptidyl-propyl isomerase cyclophilin A was able to interact with Vpr via prolines in position 14 and 35, which insured the correct folding of the viral protein [17]. The carboxy-terminus of Vpr contains six arginines between residues 73 and 96. This domain shows similarity with those of arginine-rich protein transduction domains (PTD), and may explain the transducing properties of Vpr, including its ability to cross the cell membrane lipid bilayer [6,18-20].

### Vpr is packaged into virus particles

Vpr is expressed at a late stage of the virus life cycle, but it is present during the early steps of infection of target cells since it is packaged into virions released from the producing cells. The incorporation of Vpr occurs through a direct interaction with the carboxy-terminal p6^Gag ^region of the *gag*-encoded Pr55^Gag ^precursor [21-24]. While the integrity of the α-helices of Vpr is required for efficient packaging into virions [25], a leucine-rich motif found in the p6^Gag ^region of the Pr55^Gag ^precursor is directly involved in the interaction with Vpr [23,26]. After assembly and proteolytic cleavage of Pr55^Gag ^in matrix, capsid, nucleocapsid (NCp7), and p6 mature proteins, Vpr is recruited into the conical core of the virus particle [27,28] where it is tightly associated with the viral RNA [29,30]. Interestingly, Vpr displays a higher avidity for NCp7 than for the mature p6 protein [23,24,31]. Since p6 is excluded from the virion core [27,28], Vpr could switch from the p6^Gag ^region of the precursor to the mature NCp7 protein to gain access to the core of the infectious virus particle budding at the cell surface. It seems that Vpr is less avid for the fully processed p6 protein than for the p6^Gag ^region in the context of the p55^Gag ^precursor. Because of this differential avidity, Vpr is recruited into to the core of the particle where it could interact with nucleic acids, NCp7 [24,31] and/or the matrix protein [32]. Since it was estimated that Vpr is efficiently incorporated with a Vpr/Gag ratio of ~1:7 [33], that may represent 275 molecules of Vpr per virion.

The incorporation of Vpr has been also used as a unique tool to target cargoes (i.e., cellular and viral proteins, drugs) into viral particles [34,35]. This property was extensively used to study the respective functions of integrase (IN) and reverse transcriptase (RT) during virus replication by expressing Vpr-IN and Vpr-RT fusions *in trans *in virus-producing cells [36-38]. This strategy of *trans*-complementation also allowed the analysis of mutant of IN without altering assembly, maturation and other subsequent viral events [37,39].

Furthermore, Vpr fused to the green fluorescence protein (GFP) has been recently used to tag HIV particles in order to follow intracellular virus behavior during the early steps of infection of target cells [40,41].

### Vpr influences the fidelity of the reverse transcription process

Following virus entry, the viral core is released into the cytoplasm of the target cell and the reverse transcription of the viral RNA takes place in the cytoplasm within a large nucleoprotein complex termed the reverse transcription complex (RTC) containing the two copies of viral RNA and the viral proteins: RT, IN, NCp7, Vpr and a few molecules of the matrix protein [42-46]. It is generally believed that the reverse transcription process is initiated in virus particles and is then completed, after virus entry, in the cystosol of the target cell. This process is likely concomitant of both virus uncoating and trafficking through the cytosol (for reviews, see [47,48]). Recent studies confirmed that Vpr co-localizes with viral nucleic acids and IN within purified HIV-1 RTCs [41,45,49], and remains associated with the viral DNA within 4 to 16 h after acute infection [43].

In addition to a potential role in the initiation step of the reverse transcription process [50], it has been shown that Vpr modulates the *in vivo *mutation rate of HIV-1 by influencing the accuracy of the reverse transcription. The HIV-1 RT is an error-prone RNA dependant DNA polymerase, and quantification of the *in vivo *rate of forward virus mutation per replication cycle revealed that the mutation rate was as much as fourfold higher in the absence of Vpr expression when measured in actively dividing cells using a genetically engineered system [51,52]. Furthermore, recent analysis in non-dividing cells shows that this phenotype is exacerbated in primary monocyte-derived macrophages (MDM) leading to a 18-fold increase of the HIV-1 mutation frequency [53]. This activity strikingly correlates with the interaction of Vpr with the nuclear form of uracil DNA glycosylase (UNG2) [54], an enzyme involved in the base excision repair pathway that specifically removes the RNA base uracil from DNA. Uracil can occur in DNA either by misincorporation of dUTP or by cytosine deamination. Initially identified from a yeast two-hybrid screening using Vpr as a bait, the interaction with UNG was confirmed both *in vitro *and *ex vivo *in Vpr-expressing cells. While the Trp residue in position 54 located in the exposed loop connecting the second and the third α-helix of HIV-1 Vpr has been shown critical to maintain the interaction with UNG, the Vpr-binding site was mapped within the C-terminal part of UNG2 and occurs through a TrpXXPhe motif. Currently, three distinct cellular partners of Vpr contain a WXXF motif including the TFIIB transcription factor, the adenosine-nucleotide translocator (ANT) and UNG2 [55,56].

The association of Vpr with UNG2 in virus-producing cells allows the incorporation of a catalytically active enzyme into HIV-1 particles where UNG2 may directly influence the reverse transcription accuracy [54], and this plays a specific role in the modulation of the virus mutation rate. The model supporting the direct contribution of incorporated UNG2 in the reverse transcription process was recently demonstrated by using an experimental system in which UNG2 was recruited into virions independently of Vpr. UNG2 was expressed as a chimeric protein fused to the C-terminal extremity of the VprW54R mutant, a Vpr variant that fails to recruit UNG2 into virions and to influence the virus mutation rate, even though it is incorporated as efficiently as the wild type (wt) Vpr protein. The VprW54R-UNG fusion is also efficiently packaged into HIV-1 virions and restores a mutation rate equivalent to that observed with the wt Vpr, both in actively dividing cells and in MDMs. In agreement with this phenotype on the virus mutation frequency, it was finally documented that the Vpr-mediated incorporation of UNG2 into virus particles contributes to the ability of HIV-1 to replicate in primary macrophages. When the VprW54R variant was introduced into an infectious HIV-1 molecular clone, virus replication in MDMs was both reduced and delayed whereas replication in PBMC was not altered by the lack of UNG2 incorporation into virus particles. Although it was proposed that the viral integrase was also able to mediate interaction with UNG2, Vpr seems the main viral determinant that allows for the incorporation of cellular UNG2 into virus particles. However, preliminary results obtained from *in vitro *binding assays suggest that both Vpr and IN associate with UNG to form a trimeric complex (ELR and SB, unpublished results), but further analyses are required to document the nature of the interactions between UNG2, Vpr, IN as well as RT both in virus-producing cells and then in target cells.

HIV-1 and other lentiviruses are unusual among retroviruses in their ability to infect resting or terminally differentiated cells. While Vpr has been shown to facilitate the nuclear import of viral DNA in non-dividing cells, the virion incorporation of UNG2 via Vpr also contributes to the ability of HIV-1 to replicate in primary macrophages. This implies that UNG2 is a cellular factor that plays an important role in the early steps of the HIV-1 replication cycle (i. e. viral DNA synthesis). This observation is in good agreement with a recent report showing that the misincorporation of uracil into minus strand viral DNA affects the initiation of the plus strand DNA synthesis *in vitro *[57]. This observation suggests that UNG is likely recruited into HIV-1 particles to subsequently minimize the detrimental accumulation of uracil into the newly synthesized proviral DNA. While further work is needed to explain the precise mechanism for how UNG catalytic activity may specifically influence HIV-1 replication in macrophages, it is worth noting that nondividing cells express low levels of UNG and contain relatively high levels of dUTP [58]. Similarly, most non-primate lentiviruses, such as feline immunodeficiency virus (FIV), caprine-arthritis-encephalitis virus (CAEV) and equine infectious anemia (EIAV), have also developed an efficient strategy to reduce accumulation of uracil into viral DNA. These lentiviruses encode and package a dUTP pyropshophatase (dUTPase) into virus particles, an enzyme that hydrolyzed dUTP to dUMP, and thus maintains a low level of dUTP. Interestingly, replication of FIV, CAEV or EIAV that lack functional dUTPase activity is severely affected in nondividing host cells (e.g., primary macrophages). Taken together, these results indicate that uracil misincorporation in viral DNA strands during reverse transcription is deleterious for the ongoing steps of the virus life cycle. The presence of a viral dUTPase or a cellular UNG will prevent these detrimental effects for replication of non-primate and primate lentiviruses in macrophages, respectively.

In addition, it is intriguing to note that two viral auxiliary proteins from HIV-1, Vpr and Vif, can both influence the fidelity of viral DNA synthesis. The Vif protein forms a complex with the cellular deaminase APOBEC-3G (CEM15) preventing its encapsidation into virions [59-63], while Vpr binds the DNA repair enzyme, UNG, to recruit it into the particles. It is tempting to speculate that the action of both viral proteins may influence the mutation rate during the course of HIV-1 infection, and their balance may play a key role during disease progression in infected individuals.

### Vpr and the nuclear import of the viral pre-integration complex

Nondividing cells, such as resting T cells and terminally-differentiated macrophages, are important targets for viral replication during the initial stages of infection, since primary infection of these cell populations contributes to the establishment of virus reservoirs, crucial for subsequent virus spread to lymphoid organs and T-helper lymphocytes [64]. Infection of lymphoid histoculture using human tonsil or splenic tissue showed that Vpr greatly enhances HIV replication in macrophages but did not influence productive infection of proliferating or resting T cells [65]. After virus entry into the cell, the viral capsid is rapidly uncoated and the reverse transcription of the genomic HIV-1 RNA leading to the full length double-strand DNA is completed. This viral DNA associates with viral and host cell proteins into the so-called pre-integration complex (PIC). In contrast to oncoretroviruses which require nuclear envelope disintegration during mitosis to integrate their viral genome into host chromosomes, lentiviruses, such HIV and SIV, have evolved a strategy to import their own genome through the envelope of the interphasic nucleus via an active mechanism 4–6 h after infection (for review, see [66]). Vpr has been reported to enhance the transport of the viral DNA into the nucleus of nondividing cells [67-69], by promoting direct or indirect interactions with the cellular machinery regulating the nucleo-cytoplasmic shuttling [70-74].

#### PIC en route to the NE

The exact composition of the PIC is still an area of debate but it contains the viral DNA at least associated with integrase, and many recent studies have confirmed that Vpr is also an integral component of this complex (for reviews, see [75-77]). Of course, the PIC likely contains cellular factors that participate in both intra-cytoplasmic routing and nuclear translocation of the viral DNA. While actin microfilaments seem to play a role in the early events of infection by acting as a scaffold for the appropriate localization and activation of the RTC [78], the PIC is tightly associated with microtubular structures in the cytoplasm. An elegant system using Vpr fused to GFP as a probe was developed to follow the movement of the PIC soon after virus entry in living cells [40]. It has been shown that the GFP-Vpr labeled-PIC progresses throughout the cytoplasm along cytoskeletal filaments and then accumulates in the perinuclear region close to centrosomes. More precisely, it was observed that the viral complex uses the cytoplasmic dynein motor to travel along the microtubule network to migrate towards the nucleus. It is not yet known whether Vpr plays an active role during this movement of the PIC along microtubules or whether it is only associated with the complex and then actively participates in the subsequent steps, including the anchoring of the PIC to the nuclear envelope (NE) and the nuclear translocation of the viral DNA.

#### Vpr docks at the NE

Indeed, Vpr displays evident karyophilic properties and localizes in the nucleus, but a significant fraction is anchored at the NE and can be visualized as a nuclear rim staining in fluorescence microscopy experiments [73,79-81]. The NE consists of two concentric inner and outer membranes studded with nuclear pore complexes (NPC) that form a conduit with a central aqueous channel which allows selective trafficking between the nucleus and cytoplasm and creates a permeability barrier to free diffusion of macromolecules or complexes. NPC corresponds to a 125-MDa structure consisting of 30 distinct nuclear pore proteins, named nucleoporins (Nups) [82]. A specific subset of Nups contain FG- or FxFG peptide repeats that constitute most of the filamentous structures emanating from both sides of the NPC and that provide docking sites for various transport factors [83]. Initial studies revealed that HIV-1 Vpr bound to the FG-rich region of several nucleoporins including the human p54 and p58 Nups, the rodent POM121, and the yeast NUP1P [71,73,74], but a direct interaction with the human CG1 nucleoporin was more recently reported [70]. This interaction is not mediated by the FG-repeat region of this Nup but rather via a region without consensus motif located in the N-terminus of the protein. Using an *in vitro *nuclear import assay, it has been demonstrated that the association with the N-terminal region of hCG1 is required for the docking of Vpr to the NE, whereas the FG-repeat region does not participate in this process [70]. The role of Vpr at the NE is not clear but two explanations can be proposed. First, this localization may account for the targeting of the PIC to the NPC before its translocation into the nuclear compartment. In this model, the virion-associated Vpr would be primarily involved, after virus entry and uncoating, in the initial docking step of the viral DNA to the NPC, while other karyophilic determinants of the PIC, such as IN, would then allow for the second step of nuclear translocation to proceed [81,84-86]. Alternatively, another explanation may come from the observation that Vpr was able to provoke herniations and transient ruptures of the NE [87]. The molecular mechanism supporting the local bursting induced by Vpr is not known but the interaction of Vpr with nucleoporins may cause initial misassembly of the NPC leading to alterations of the NE architecture. Consequently, these transient ruptures may provide an unconventional route for nuclear entry of the viral PIC [87,88].

#### Translocation of Vpr into the nucleus

Despite the lack of any identifiable canonical nuclear localization signal (NLS), Vpr displays evident karyophilic properties and is rapidly targeted to the host cell nucleus after infection [89]. Even though the small size of Vpr does not strictly require an NLS-dependent process, experiments performed both *in vitro *or in transfected cells have shown that Vpr is able to actively promote nuclear import of a reporter protein, such as BSA, β-galastosidase or GFP [10,13,90-94]. Like proteins containing a basic-type NLS, it was initially proposed that Vpr uses an importin α-dependant pathway to access the nuclear compartment [72,73]. In addition, Vpr may enhance the inherently low affinity of the viral MA for importin α to allow nuclear import of MA [95,96], but conflicting data exists on the nuclear localization of this viral protein [81,85]. Finally, it was reported that Vpr nuclear import was mediated by an unidentified pathway, distinct from the classical NLS- and M9-dependant pathways [92]. Two independent nuclear targeting signals have been characterized within the HIV-1 Vpr sequence, one spanning the α-helical domains in the N-terminal part of the protein and the other within the arginine-rich C-terminal region [92,94]. These results are consistent with data showing that the structure of the α-helical domains of Vpr must be maintained both for its nuclear localization and for Vpr binding with nucleoporins [25,70,80].

In conclusion, the nucleophilic property of Vpr and its high affinity for the NPC, associated with its presence in the viral PIC, at least support a role during the docking step of the PIC at the NE, a prerequisite before the translocation of viral DNA into the nucleus. Even though there is no evidence that Vpr directly participates in the translocation process, it is worth noting that purified PICs also dock at the NE before nuclear translocation using a pathway also distinct from the NLS and M9 nuclear import pathways [49]. One can suggest that among the redundancy of nuclear localization signals characterized within the PIC, both in associated viral proteins (i.e. IN, MA, Vpr) and also in the viral DNA [97], Vpr primarily serves to dock the PIC at the NE, while IN and MA act in cooperation with the central DNA flap to target the viral DNA to the nucleus (for review, see [98]).

#### Vpr, a nucleocytoplasmic protein

In addition to its nonconventional NLS for targeting into the nucleus, Vpr is a dynamic mobile protein able to shuttle between the nucleus and cytoplasmic compartments [23,99,100]. Photobleaching experiments on living cells expressing a Vpr-GFP fusion confirmed that Vpr displays nucleocytoplasmic shuttling properties [70]. This shuttling activity has been related to the distal leucine-rich helix which could form a classical CRM1-dependant nuclear export signal (NES) [99]. The exact role of this NES in the function of Vpr is not known but since Vpr is rapidly imported into the nucleus after biosynthesis, the NES could redirect it into the cytoplasm for subsequent incorporation into virions through direct binding to the viral p55^Gag ^precursor during the late budding step of the virus life cycle [23,100].

### Vpr and the cell cycle

A further important biological activity of SIV and HIV Vpr proteins is related to their ability to induce an arrest in the G2 phase of the cell cycle of infected proliferating human and simian T cells [91,101-105]. Cell cycle arrest does not require de novo synthesis of Vpr, but is induced by Vpr molecules packaged into infecting virions [87,106]. This indicates that induction of the G2 cell cycle arrest might happen before the integration step of the viral DNA genome. It is noteworthy that the *S. pombe *fission yeast as well as *S. cerevisiae *overexpressing HIV-1 Vpr are also blocked in the G2 phase of the cell cycle [107-109], supporting the idea that the cellular pathway altered by Vpr is well conserved in all eukaryotic cells. Moreover, infection of caprine cells with a caprine arthritis encephalitis virus (CAEV) expressing the *vpr *gene from SIV similarly provoked a G2 arrest [110]. The biological significance of this arrest during the natural infection is not well understood, but the HIV-1 LTR seems to be more active in the G2 phase, implying that the G2 arrest may confer a favorable cellular environment for efficient transcription of HIV-1 [111]. In agreement, the Vpr-induced G2 arrest correlates with high level of viral replication in primary human T cells.

The determinants of the G2 arrest activity are mainly located in the C-terminal unstructured basic region of HIV-1 Vpr and phosphorylation of the protein is required [112,113]. Regulators of the cell cycle, such as cyclin-dependant kinases (CDKs), control progression through the cell cycle by reversible phosphorylation [114]. The p34/cdc2 CDK associates with cyclin B1 in the G2 phase (for review, see [115]) to regulate the G2 to M transition. Accumulation of the cells expressing Vpr in the G2 phase has been correlated to the inactivation of the p34/cdc2-cyclinB kinase [102,103]. The activity of cdc2 is controlled by opposite effects of the Wee-1 and Myt1 kinases and the cdc25 phosphatase. Wee1 inhibits cdc2 activity through tyrosine phosphorylation, while dephosphorylation of cdc2 by the phosphatase cdc25 promotes cdc2-cyclinB activation that drives cells into mitosis. The activities of both cdc25 and Wee-1 are also regulated by phosphorylation/dephosphorylation. It was initially described that Vpr-expressing cells contained both hyperphosphorylated cdc2 and hypophosphorylated cdc25, their inactive status [101-103]. Consequently, these two regulators of the G2/M switch are blocked preventing any cell cycle progression. The molecular mechanism leading to this inhibition is not yet clear, but different cellular partners interacting with Vpr which could play a role in cell cycle regulation have been proposed as potential mediators of the Vpr-induced G2 arrest. hVIP/MOV34, a member of the eIF3 complex, was identified as a Vpr-partner in a yeast two-hybrid assay [116], and was associated with the cell cycle arrest activity of Vpr [117]. eIF3 is a large multimeric complex that regulates transcriptional events and is essential for both G1/S and G2/M progression. Intracellular localization studies revealed that expression of Vpr induces a relocalization of MOV34 that shifts from a cytoplasmic to a nuclear localization pattern [116,117]. Two other cellular partners of Vpr, UNG and HHR23A (i.e., the human homologue of the yeast rad23 protein), are implicated cellular DNA repair processes. Since a clear relationship exists between the DNA damage response pathway and the progression of the cell cycle, it was initially suggested that Vpr binding to these DNA repair proteins could account for the observed G2 arrest [118-120], but subsequent analyses indicated that there was no correlation between the association of Vpr with HHR23A and/or UNG and the block in G2 [121,122]. These analyses are in agreement with a previous report showing that the Vpr-mediated arrest is distinct from the cell cycle arrest in G2 related to DNA damage. However, it has also been reported that Vpr induces cell cycle arrest via a DNA damage-sensitive pathway [123]. The G2 DNA damage checkpoint is under the control of the phosphatidylinositol 3-kinase-like proteins, ATR and ATM [124], which lead to the inactivation of the cdc2-cyclinB complex. The ATR protein has been recently linked to the G2-arrest induced by Vpr [125]. Inhibition of ATR either by drugs, a dominant-negative form of ATR or by siRNA reverts the Vpr-induced cell cycle arrest while activation of ATR by Vpr results in Chk1 phosphorylation, the kinase regulating cdc25c activity. These authors suggested that the G2 arrest induced by Vpr parallels the ATR-DNA damage pathway, but additional work is needed to demonstrate that Vpr causes DNA damage or mimics a signal activating one of the DNA damage sensors.

The protein phosphatase 2A (PP2A) has been shown to be directly associated with Vpr via its B55α subunit [126]. PP2A is a serine/threonine phosphatase involved in a broad range of cellular processes, including cell cycle progression. PP2A inactivates cdc2 indirectly both by the inactivation of the Wee1 kinase and by activation of cdc25 (for review, see [127]). Genetic studies performed in *S. pombe *suggest the involvement of PP2A and Wee1 in the Vpr-induced cell cycle arrest [128]. Intriguingly, expression of Vpr and B55α results in the nuclear localization of B55α subunit while it remains cytoplasmic in normal condition. Together, these studies emphasized the fact that Vpr might play a role in the subcellular redistribution of several regulatory protein complexes involved in the progression of the cell cycle. Indeed, the mitotic function of cdc2-cyclinB complex is triggered not only by the turn of phosphorylation/desphorylation of both subunits on specific residues, but also by spatio-temporal control of their intracellular distribution. For example, cyclinB is predominantly cytoplasmic throughout the G2 phase until it translocates rapidly into the nucleus 10 min before nuclear envelope breakdown [129]. As mentioned earlier, Vpr induces herniations and local bursting of the nuclear envelope leading to redistribution of key cell cycle regulators, including Wee1, cdc25, and cyclin B into the cytoplasm of the host cell [87]. It seems evident that alterations of the subcellular localization of segregated cell cycle regulators could explain the G2 arrest induced by Vpr; this may also explain the overall variety of cellular factors that have been involved in this process. Alternatively, nuclear herniations induced by Vpr could also affect chromatin structure leading to the activation of ATR. However, it not known if the Vpr-induced alteration of the NE architecture could cause DNA damage such as double-strand breaks, but disruption of the nuclear lamin structure is sufficient to block DNA replication, another abnormality recognized by the ATR protein (for reviews, see [130,131]).

### Vpr and apoptosis

HIV infection causes a depletion of CD4^+ ^T cells in AIDS patients, which results in a weakened immune system, impairing its ability to fight infections. The major mechanism for CD4^+ ^T cell depletion is programmed cell death, or apoptosis, that can be induced by HIV through multiple pathways of both infected cells and non-infected "bystander" cells (for review, see [132]). Even though the exact contribution of Vpr as a pro-apoptotic factor responsible for the T cell depletion observed in the natural course of HIV infection is still unknown, it was repeatedly evidenced that Vpr has cytotoxic potential and is able to induce apoptosis in many *in vitro *systems. In addition, transgenic mice expressing Vpr under the control of the CD4 promoter show both CD4 and CD8 T cell depletion associated with thymic atrophy [133]. However, controversial results indicating that Vpr can also act as negative regulator of T cell apoptosis have been reported [134,135].

Initially proposed as a consequence of the prolonged cell cycle arrest [136-140], other investigations have then revealed that the Vpr-mediated G2 arrest was not a prerequisite for induction of apoptosis, suggesting that both functions are separated [79,87,141,142]. However, the recent observation that the activity of the cell cycle regulatory Wee-1 kinase is decreased in Vpr-induced apoptotic cells led to the hypothesis of a direct correlation between the G2 arrest and apoptotic properties of Vpr [143]. Hence, reduction of Wee-1 activity, probably related to its delocalization provoked by Vpr [87], results in an inappropriate activation of cdc2 leading to cell death with phenotypical aberrant mitotic features, a process known as mitotic catastrophe [144,145]. Using an established cell line expressing Vpr, it was observed that after the long G2 phase, cell rounded up with aberrant M-phase spindle with multiple poles resulting from abnormal centrosome duplication [138,146]. The cells stopped prematurely in pro-metaphase and died by subsequent apoptosis.

However, works from the G. Kroemer's group have then well established that synthetic Vpr, as well as truncated polypeptides, are able to induce apoptosis by directly acting on mitochondria leading to the permeabilization of the mitochondrial membrane and subsequent dissipation of the mitochondrial transmembrane potential (ΔΨm) [56]. This direct effect of Vpr was related to its ability to interact physically with the adenine nucleotide translocator (ANT), a component of the permeability transition pore of mitochondria localized in the inner mitochondrial membrane. Since ANT is a transmembrane protein and presents a WxxF motif on the inner membrane face which is recognized by Vpr [56,147], this interaction implies that Vpr must first cross the outer mitochondria membrane to access ANT. The interaction between Vpr and ANT triggers permeabilization of the inner membrane followed by permeabilization of the outer mitochondrial membrane with consequent release of soluble intermembrane proteins, such as cytochrome *c *and apoptosis inducing factors, in the cytosol. Cytochrome *c *then associates with Apaf-1 in a complex with caspase-9 to create the apoptosome, allowing activation of effector caspases, such as caspase-3, and subsequently the final execution of the apoptotic process (for review, see [148]). While numerous reports have shown that Vpr mediated-apoptosis was associated with activation of caspase-9 and capase-3 [56,79,137,140,147,149], it is intriguing that Vpr was still able to induce cell death in embryonic stem cells lacking Apaf-1, caspase-9 and IAF [150]. These results suggest a model in which the direct action of Vpr on mitochondria may be sufficient to cause cell death in HIV-1 infected cells [149].

Although the causal role of Vpr in the induction of apoptosis is evident both *in vitro *and *ex vivo*, its real contribution with other viral determinants, such as gp120 envelope, Tat, Nef and the viral protease, in the physiopathology of AIDS needs to be further documented during the course of HIV infection [151]. However, it was recently revealed that long term non-progressor HIV-1 infected patients show a highest frequency of mutation at the position Arg77 of the Vpr protein than patients with progressive AIDS disease. Interestingly, this residue seems crucial for the capacity of the protein to induce apoptosis through permeabilization of the mitochondrial membrane [152]. Conversely, it was reported that mutation of the Leu64 residue enhanced the pro-apoptopic activity of Vpr [153], indicating that mutations affecting the C-terminal region of the protein may generate Vpr molecules with different pro-apoptotic potentials during the course of natural HIV-1 infection.

In addition, soluble Vpr protein is found in the sera as well as in the cerebrospinal fluid of HIV-infected patients, and was proposed to play a role related to its pro-apoptotic activity in AIDS-associated dementia [154,155]. The involvement of Vpr in these neurological disorders has been suggested, since recombinant Vpr has neurocytopathic effects on both rat and human neuronal cells [156-158]. Neurons killed by extracellular Vpr display typical features of apoptosis evidenced by direct activation of the initiator caspase-8 that will lead to subsequent activation of effector caspases. These effects have been linked to the property of the first amphipathic α-helix of Vpr to form cation-selective ion channels in planar lipid bilayers, causing a depolarization of the plasma membrane [6,157,159,160]. These observations indicate that Vpr can trigger apoptotic processes by different alternative pathways depending of the target cells.

### Nuclear role(s) of Vpr

The first reported function of Vpr was a modest transcriptional activity on the viral LTR promotor as well as on heterologous cellular promotors [161,162]. While the connection between cell cycle arrest and LTR-transactivation by Vpr is not well understood, it was concluded that activation of the Vpr-induced viral transcription is secondary to its G2/M arrest function [111,163]. An increase transcriptional activity is indeed observed from the viral LTR in arrested cells expressing Vpr [164-166]. The transactivation of HIV-1 induced by Vpr is mediated through *cis*-acting elements, including NF-κB, Sp1, C/EBP and the GRE enhancer sequences found in the LTR promotor [167-170]. Also related to this activity, Vpr regulates the expression of host cell genes such as NF-κB, NF-IL-6, p21^Waf1 ^and *survivin *[171-173]. Finally, Vpr seems also able to interact directly with the ubiquitous cellular transcription factor Sp1 [168], the glucocorticoid receptor [174,175], the p300 coactivator [163,176], and with the transcription factor TFIIB, a component of the basal transcriptional machinery [177]. This latter interaction is also mediated by a WxxF motif found within the TFIIB primary sequence [55].

Vpr displays high affinity for nucleic acids but no specific DNA sequence targeted by Vpr has been yet identified [19,29]. Interestingly, Vpr does not bind to the Sp1 factor or *cis*-acting elements alone but it associates with Sp1 in the context of the G/C box array [168], as well as in a ternary complex with p53 [178], indicating that Vpr might bind specific DNA sequence once associated with cellular partners to subsequently drive expression of both host cell and viral genes. Consistently, it has been reported that Vpr can directly bind to p300 via a LXXLL motif present in the C-terminal α-helix of the protein [179], suggesting that Vpr may act by recruiting the p300/CBP co-activators to the HIV-1 LTR promotor and thus enhance viral expression. Since p300 is a co-activator of NF-κB, Vpr can also mediate up-regulation of promotors containing NF-κB and NF-IL-6 enhancer sequences in primary T cells and macrophages. In addition, Vpr markedly potentiates glucocorticoid receptor (GR) action on its responsive promotors [174,175]. The Vpr-mediated LTR transcription was inhibited by the addition of the GR antagonist, RU486, in cultured macrophages [175]. That Vpr-mediated co-activation of the GR is distinct from the G2 arrest and required both LLEEL^26 ^and LQQLL^68 ^motifs contained within the first and third α-helical domains of HIV-1 Vpr [174,180].

Vpr may also function as an adaptor molecule for an efficient recruitment of transcriptional co-activators (GRE, p300/CBP...) to the HIV-1 LTR promotor and thus enhances viral replication. Additionally, it may be involved in the activation of host cell genes inducing cellular pathways in relation with the AIDS pathogenesis. Indeed, cDNA microarray analysis using isogenic HIV-1 either with or without *vpr *expression revealed that Vpr induces up and down regulation of various cell genes [181].

## Conclusion

By interfering with many distinct cellular pathways all along the virus life cycle, it is now evident that Vpr's contribution to the overall pathogenesis of HIV-1 infection *in vivo *is likely crucial. While major efforts have been made during the last years to define the molecular mechanisms and cellular targets of Vpr, additional work is needed for the complete understanding of its wide range of activities. An important issue now is to define the precise contribution of each activity to the viral replication and pathogenesis during the natural course of HIV infection. The involvement of Vpr in key processes of the early steps the viral life cycle (i.e., reverse transcription and nuclear import of the viral DNA) represents a good target for developing novel therapeutic strategies for AIDS therapy. In addition, this viral factor represents a valuable tool to elucidate many fundamental cellular processes.

## List of abbreviations

HIV, human immunodeficiency virus; SIV, simian immunodeficiency virus; CypA, cyclophilin A; nup, nucleoporin; PIC, pre-integration complex; RTC, reverse transcription complex.
